# Development of a sensitive non-radioactive protein kinase assay and its application for detecting DYRK activity in *Xenopus laevis *oocytes

**DOI:** 10.1186/1471-2091-11-20

**Published:** 2010-05-20

**Authors:** Eva Lilienthal, Katharina Kolanowski, Walter Becker

**Affiliations:** 1 Institute of Pharmacology and Toxicology, Medical Faculty of the RWTH Aachen University, Wendlingweg 2, 52074 Aachen, Germany

## Abstract

**Background:**

Although numerous non-radioactive methods are in use to measure the catalytic activity of protein kinases, most require specialized equipment and reagents and are not sufficiently sensitive for the detection of endogenous kinase activity in biological samples. Kinases of the DYRK family have important functions in developmental and pathophysiological processes in eukaryotic organisms including mammals. We aimed to develop a highly sensitive, low-tech assay suitable to determine the activity of DYRK family kinases in tissues or cells from diverse sources.

**Results:**

Phosphorylation-site specific antibodies can be used to monitor the accumulation of the phosphorylated product in kinase assays. We present a modified configuration of an enzyme-linked immunosorbent assay (ELISA)-based kinase assay by using the phosphospecific antibody as the capture antibody. This assay format allowed the detection of small amounts of phosphopeptide in mixtures with an excess of the unphosphorylated substrate peptide (10 fmol phosphorylated peptide over a background of 50 pmol unphosphorylated peptide). Consequently, low substrate turnover rates can be determined. We applied this method to the measurement of endogenous DYRK1A activity in mouse heart tissue by immunocomplex kinase assay. Furthermore, we detected DYRK1-like kinase activity in *Xenopus laevis *oocytes and identified this kinase as a DYRK1 isoform distinct from the *Xenopus *DYRK1A ortholog.

**Conclusion:**

We present a non-radioactive and highly sensitive method for the measurement of endogenous activities of DYRKs in biological samples. *Xenopus laevis *oocytes contain an active DYRK1-related protein kinase more similar to mammalian DYRK1B than DYRK1A.

## Background

Most cellular processes are controlled by protein phosphorylation, and aberrant kinase activity has been implicated in the etiology of a wide spectrum of diseases, including cancer, chronic inflammatory disorders and neurodegeneration. Studies on protein kinases are important not only to elucidate molecular mechanisms of signal transduction, but also for drug development. Therefore, methods for measuring kinase activity and for the identification of kinase inhibitors have become increasingly important in biomedical research [1,2].

A widely employed type of assay is based on the use of radioactively labelled ATP as phosphate donor and subsequent detection of phosphate incorporation into a protein or peptide substrate that contains the respective kinase recognition motif [3,4]. This radiometric technique is simple and suitable for detection of protein kinase activity with high sensitivity but depends on the use of radioactive isotopes (^32^P or ^33^P). Use of radioactivity requires special handling, is associated with inherent high costs of waste disposal, and restricts the flexibility because of the short half life of ^32^P and ^33^P. Furthermore, these assays are carried out at subphysiological levels of ATP owing to the necessity of keeping ATP levels, and thus the usage of radioisotopes, within reasonable limits.

To circumvent these drawbacks, a wide variety of non-radiometric techniques have been developed to measure kinase activity, particularly for use in high throughput screening of kinase inhibitors (for recent reviews see [1,2]. Several non-radiometric methods rely on antibodies that can distinguish phosphorylated from unphosphorylated forms of the kinase substrates [5]. Such phosphorylation state-specific antibodies were first used by Yano et al. [6] to measure protein kinase activity by an ELISA method. In the original format, the *in vitro*-kinase reaction takes place in the wells after coating of the substrate to the surface of the microplate wells, and the phosphorylated molecules are detected with a phosphospecific antibody [6-8]. The use of biotinylated peptides allows the reaction to be performed in solution before the substrate captured on streptavidin coated plates [9,10]. An inherent drawback of the existing ELISA-based assays is that in case of low enzymatic turnover, the large amount of unphosphorylated substrate will outcompete the phosphorylated substrate for binding to the surface of the wells. This decreases the overall sensitivity of the assay, and radiometric assays are generally preferred for detecting endogenous kinase activity.

Protein kinases of the DYRK family have been implicated in a number of important biological processes in diverse eukaryotic organisms, *e.g. *Pom1p in cell morphogenesis and mitotic entry in *S. pombe *[11,12], MBK2 in oocyte maturation in *C. elegans *[13] and a DYRK1 isoform in *Xenopus laevis *oocyte maturation [14], *minibrain *(MNB) in neurogenesis in *Drosophila *[15], and DYRK1A in mammalian brain development and in neurodegeneration [16,17]. Interestingly, alterations in neuronal development were observed in mouse models both with a selective gain or partial loss of function of *Dyrk1A *(for recent reviews see [17,18]). This gene dosage effect implies that subtle changes in the activity of this DYRK family kinase can have severe consequences.

Many investigators are characterising the role of DYRKs in various biological processes or their involvement in human diseases [19-22]. For measuring the activity of DYRKs, radiometric assays are presently the standard in laboratory practice. We aimed at developing a non-radiometric assay sufficiently sensitive to measure kinase activity of endogenous DYRKs. By a modification of the existing ELISA configurations, we accomplished to reach a detection limit in the range of radiometric assays. The sensitivity of the assay was sufficient to measure the activity of DYRK1A in mouse heart. Moreover, we used the new method to characterize the activity of a DYRK1 isoform expressed in *Xenopus laevis *oocytes.

## Results

### Development and characterization of the assay

**Figure 1:**
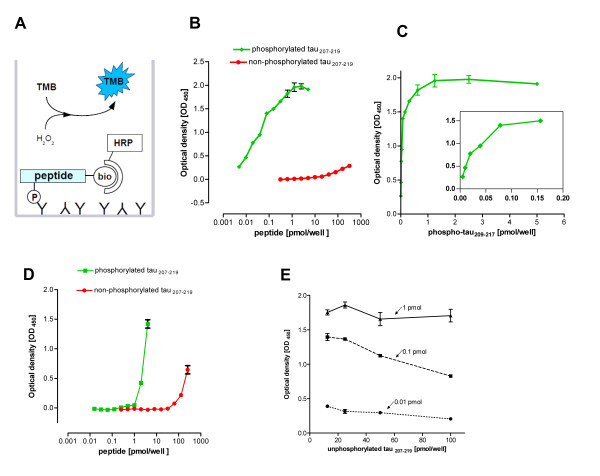
**Direct sandwich ELISA format for the detection of the phosphorylated DYRK substrate peptide tau_207-219_. A**, Scheme illustrating the principle of the assay. Bio, biotin; TMB, tetramethylbenzidine; HRP, horseradish peroxidase (coupled to streptavidin). **B **and **C**, Titration of phosphorylated and unphosphorylated tau_207-219_. The wells were coated with 100 ng anti tau(pT212) and loaded with dilution series of either phosphorylated or unphosphorylated tau_207-219_. The background signal from wells loaded only with the buffer was subtracted from all values. A representative experiment of three is shown. Panel **C **presents the same data as in panel B with a linear x-axis to visualize the linear range of the ELISA. The inset shows an enlargement of lower range. **D**, Titration of phosphorylated and non-phosphorylated tau_207-219 _on streptavidin-coated wells. Detection was performed with primary anti-tau(pT212) antibody and secondary goat anti-rabbit antibody coupled to HRP. The graph is representative of two experiments. **E**, Detection of phosphorylated tau_207-219 _in the presence of excess unphosphorylated peptide. Different amounts of phosphorylated tau_207-219 _(0.01 pmol, 0.1 pmol, 1 pmol) were mixed with a dilution series of unphosphorylated tau_207-219 _(12.5 - 100 pmol). Signals obtained in wells loaded only with the same amount of the unphosphorylated peptide were subtracted from the read-out of the mixtures. The graph is representative of two experiments. In B-E, error bars indicate the difference between duplicate wells.

Considering that the sensitivity in phospho-ELISA methods is mainly limited by the number of substrate binding sites available for immobilization, we reasoned that this problem could be overcome by using the phosphospecific antibody for capturing the small amounts of phosphopeptide from the complex reaction mixture. We decided to use a substrate peptide mimicking the sequence around Thr212 in the tau protein (also called microtubule-associated protein tau, MAPT). This is a well characterized phosphorylation site of DYRK1A [23,24], and phosphospecific antibodies directed against this site are commercially available. We used a biotinylated substrate peptide to allow for colorimetric detection of the bound phosphopeptide with the help a streptavidin-HRP conjugate (Figure 1A). Concentrations of the capture antibody and the streptavidin-HRP conjugate were optimized to develop the standard protocol used in all experiments shown here (see Methods section).

We performed a titration experiment to determine the key parameters of the assay, *i.e. *the power to discriminate between the phosphorylated and unphosphorylated biotinylated tau peptide (hereafter referred to as tau_207-219_), the absolute lower detection limit, and the linear range of the assay. As shown in Figure 1B, the detection limit was about 5-10 fmol phosphopeptide per well. A comparable signal was obtained with 320 pmol of the unphosphorylated peptide, indicating that this combination of phosphospecific antibody and substrate peptide offers excellent selectivity for the detection of the phosphorylated substrate (~ 10^5^-fold discrimination). Saturation of the assay was reached at about 1 pmol of phosphopeptide, but the linear plot (Figure 1C) illustrates that the useful measuring range was between 0.01 pmol and 0.1 pmol. Thus, the sensitivity of the ELISA compares well with radiometric assays, where about 0.1 pmol of phosphopeptide can be routinely detected. For comparison, we analysed the same concentrations of phosphorylated and unphosphorylated tau_207-219 _in the inverse configuration, in which the phosphospecific antibody was used to detect the biotinylated peptide after binding to streptavidin-coated plates (Figure 1D). This assay was much less sensitive (detection limit of 1 pmol phosphorylated peptide) and suffered from crossreaction of the antibody with the unphosphorylated peptide at concentrations greater than ~ 40 pmol per well.

Next we tested whether small amounts of the phosphopeptide can be detected in mixtures with a large excess of the unphosphorylated peptide. Although the experiment shown in Figure 1B suggested that 50-100 pmol of unphosphorylated peptide should only marginally contribute to the total signal, these concentrations reduced signal intensities obtained with 10-1000 fmol phosphopeptide (Figure 1E). We decided to apply a maximum of 50 pmol total peptide per well in subsequent experiments, and to keep this amount constant in all samples of an assay (including the standards).

### Measurement of DYRK1A kinase activity

**Figure 2:**
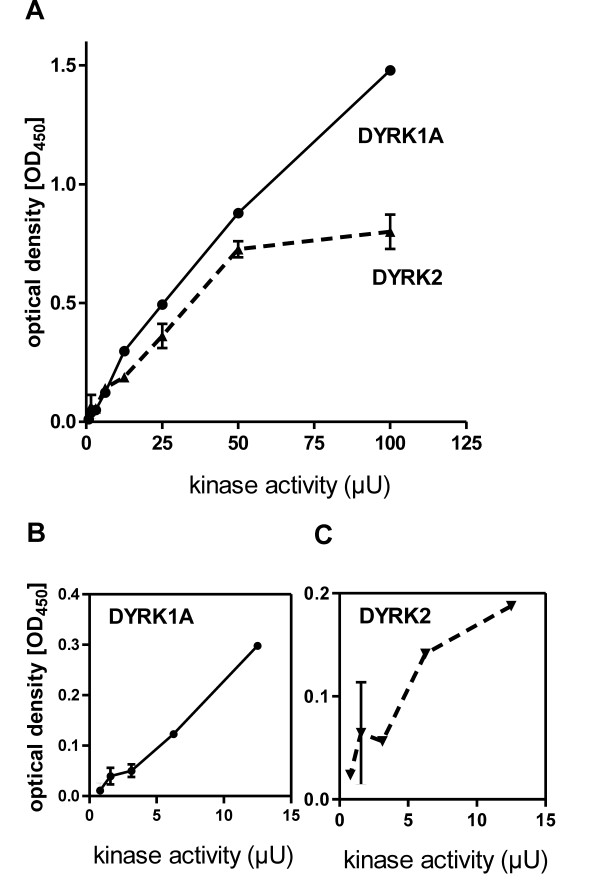
**Assay sensitivity. A**, *In vitro-*kinase reactions were performed with 50 μM tau_207-219_, 100 μM ATP and variable concentrations of GST-DYRK1A-ΔC or GST-DYRK2 for 30 min at 30°C. Reactions were stopped by addition of EDTA and peptide phosphorylation was analysed by the ELISA method. Panels **B **and **C **show enlarged views of the lower kinase concentration range. Error bars indicate the difference between duplicate measurements.

We performed *in vitro*-kinase reactions with varying concentrations of recombinant GST-DYRK1A-ΔC to determine the minimal detectable amount of kinase activity (Figure 2). The titration revealed a useable linear measuring range between 10 and 100 μU kinase activity, corresponding to 20-200 pg of the recombinant kinase. This results is in the range that could be predicted from the detection limit of the ELISA (> 10 fmol phosphopeptide), because 10 μU of kinase should phosphorylate 300 fmol substrate within 30 min, given that a sample of 1/10 of the reaction mix was loaded per well and that the excess of unphosphorylated peptide does not severely affect detection of the phosphopeptide (Figure 1E). A similar sensitivity was achieved for GST-DYRK2, consistent with the previous finding that both DYRK1A and DYRK2 can phosphorylate Thr212 in the tau protein [23].

Next we tested whether this sensitivity was sufficient to detect endogenous activity of DYRK1A immunoprecipitated from mammalian tissue. We used mouse heart for this experiment, because DYRK1A has recently been identified as a negative regulator of cardiomyocyte hypertrophy [25]. The lysate was used for parallel immunoprecipitations with a DYRK1A-specific antibody and anti FLAG antibody as a negative control. After the washing steps, the resin of each sample was split and subjected in parallel to a non-radiometric and a radiometric immunocomplex kinase assay. The activity of the bound kinase was calculated from the amount of phosphate incorporation (radiometric assay) or by comparison with a standard curve (ELISA). The results in Table 1 show that the ELISA method was sufficiently sensitive to detect endogenous DYRK1A activity in mouse heart, although background levels were somewhat higher than in the radiometric assay.

**Table 1:** DYRK1A kinase activity in mouse heart

	Phosphorylated peptide (pmol)			
		
	IP		Specific result*	relative background
			
	αDYRK1A	αFLAG		
**Radiometric assay**	5.63	1.20	4.43	21%
**ELISA**	7.88	2.24	5.63	28%

*subtraction of background (phosphorylation in αFLAG sample)

Immunoprecipitates from mouse heart lysate were split and subjected either to a radiometric or a non-radiometric kinase reaction each carried out under the same conditions (100 μM ATP, 50 μM tau_207-209_, 30 min, 30°C). The amounts of phosphorylated peptide were calculated from incorporation of ^33^P or determined by ELISA using a standard curve. As a background control, a parallel immunocomplex kinase assay was performed with anti-FLAG antibody instead of the anti-DYRK1A antibody. Aliquots of kinase reactions were diluted 1:10 for the ELISA. Results are means of duplicate measurements.

### Activity measurements of a DYRK1-related kinase in *Xenopus laevis *oocytes

DYRK kinases from distantly related organisms exhibit high sequence conservation in the catalytic domain (*e.g*. 85% identity between human DYRK1A and the *Drosophila *kinase *minibrain*) and are thus likely to recognize similar sequences in their substrates. Therefore, we reasoned that the ELISA assay established for mammalian DYRK1A could also be useful to measure DYRKs in other species. We decided to use *Xenopus *oocytes as a model system to test this assumption, because DYRK1A has been reported to play a role in oocyte maturation [14]. Database searching revealed sequences of two DYRK1 isoforms encoded by different genes (Table 2). One of these kinases shows 97% of sequence identity with human DYRK1A in the catalytic domain and can be regarded as the *Xenopus *ortholog of DYRK1A (xDYRK1A). The other one shows comparable sequence similarity with human DYRK1A and DYRK1B in the catalytic domain, but is much more similar to DYRK1B than DYRK1A in the C-terminal domain (see Additional file 1: Figure S1 for the complete sequence alignment). For the purpose of this report, we designate the latter kinase xDYRK1B.

**Table 2:** Characteristics of the two *Xenopus *DYRK1 isoforms

		
	xDYRK1A	xDYRK1B
**Database entries**		
XenBase^a ^gene symbol	dyrk1a	dyrk1a.2
UniProt^b^	Q2TAE3	Q7ZXV4
UniGene^c^	Xl.29801	Xl.5747
**Expression clones used**		
IMAGE clone	5542675	4724858
GenBank acc.	BC110968	BC044104
**Protein characteristics**		
molecular mass	84 kDa	76 kDa
histidine repeat^d^	His_11_	absent
**Sequence identity with hDYRK1A**		
catalytic domain	97%	90%
C-terminal domain	85%	<30%
**Sequence identity with hDYRK1B**		
catalytic domain	84%	87%
C-terminal domain	<30%	59%

^a ^http://www.xenbase.org; ^b ^http://www.uniprot.org^c ^http://www.ncbi.nlm.nih.gov/unigene

^d ^Human DYRK1A contains a repeat of 13 consecutive histidines, human DYRK1B contains no histidine repeat

**Figure 3:**
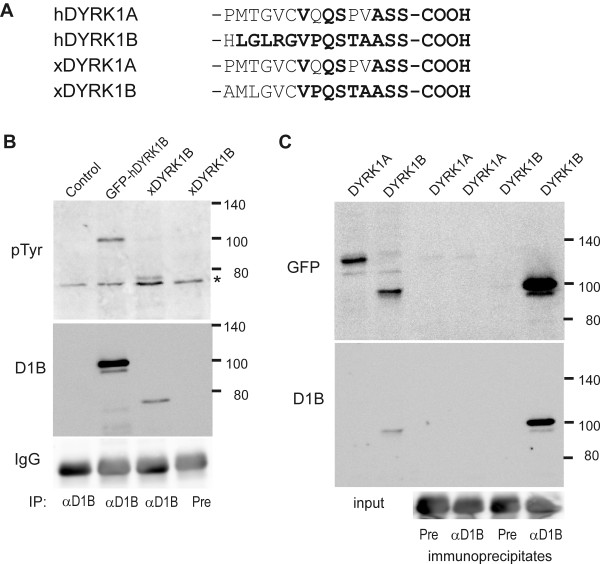
**Immunoprecipitation of xDYRK1B**. **A**, C-terminal sequences of human and *Xenopus *DYRK1A and DYRK1B. Amino acids identical with the immunogenic peptide used to raise the DYRK1B antibody are highlighted in bold. **B**, Immunoprecipitation of xDYRK1B. HeLa cells were transiently transfected with expression plasmids for xDYRK1B, GFP-DYRK1B or the empty vector (*control*). After immunoprecipitation with DYRK1B-specific antiserum (*αD1B*) or preimmune serum (*Pre*), bound proteins were subjected to Western blot analysis with a phosphotyrosine-specific antibody (*P-Tyr*) and the DYRK1B antiserum as indicated. Detection of IgG is shown as a loading control. The asterisk marks an unspecific band. **C**, Specificity of the DYRK1B antiserum. COS7 cells were transiently transfected with expression plasmids for the GFP fusion proteins of mammalian DYRK1A or DYRK1B as indicated on top of the lanes. Aliquots of the whole cell lysates (*input*) or proteins eluted from the immunoprecipitates (*αD1B, Pre*) were subjected to Western blot analysis with anti GFP and anti DYRK1B antibodies as indicated. Detection of IgG is shown as a loading control.

The mRNA originally detected in *Xenopus *oocytes by Qu *et al. *[14] corresponds to xDYRK1B (see discussion). To immunoprecipitate this kinase, we took advantage of a polyclonal antiserum we had previously raised against a peptide with the sequence of the 15 C-terminal amino acids of mouse DYRK1B [26], of which 9 are identical with xDYRK1B (Figure 3A). To test whether this antiserum also recognized xDYRK1B, we overexpressed xDYRK1B in mammalian cells. As shown in Figure 3B, the antiserum was indeed suitable for immunoprecipitation and subsequent immunodetection of recombinant xDYRK1B by Western blot analysis. No band was detected in a control immunoprecipitation with serum taken from the same rabbit before immunization. The immunopurified protein contained immunodetectable phosphotyrosine, a common feature of the members of the DYRK family. Taking advantage of the fact that mammalian and *Xenopus *DYRK1A have identical sequences in the region corresponding to the immunogenic peptide (Figure 3A), we overexpressed GFP-DYRK1A in Hela cells to assess whether the DYRK1B antiserum might crossreact with xDYRK1A. Importantly, anti DYRK1B did not immunoprecipate GFP-DYRK1A (Figure 3C), implying that it specifically immunoprecipitates xDYRK1B and not xDYRK1A from the oocytes.

**Figure 4:**
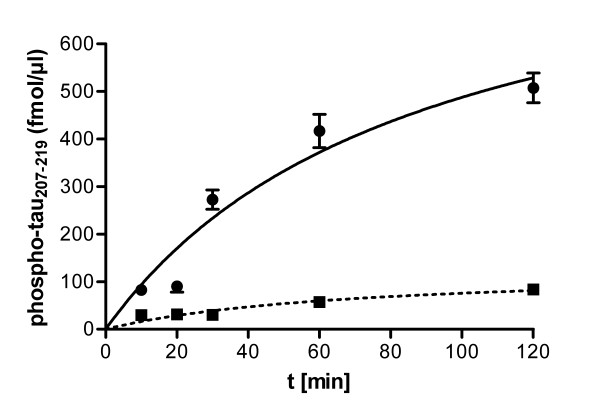
**Immunocomplex kinase assay of xDYRK1B from *Xenopus laevis *oocytes**. *Xenopus *oocytes were lysed and the lysate was subjected to immunoprecipitation with either DYRK1B antiserum or preimmune serum (*αD1B, Pre*). Aliquots from immunocomplex kinase reactions (100 μM ATP, 50 μM tau_207-219_, 30°C, total volume of 50 μL) were taken at different times and the amounts of phosphorylated tau_207-219 _were determined by the ELISA method with the help of a standard curve. Error bars represent the difference between the duplicate measurements.

We used the DYRK1B antibody for immunocomplex kinase assays to determine whether unstimulated *Xenopus *oocytes contained active xDYRK1B. First we determined the phosphorylation of tau_207-219 _by the immunoprecipitates over time (Figure 4). Kinase activity was well detectable in the anti DYRK1B immunoprecipitates, with little background in the preimmune serum control. We decided to use an incubation time of 30 min for the following assays because the turnover was flattening after this time point. The detection of xDYRK1B activity was reproduced in three independent assays with different batches of oocytes and was also confirmed in a radiometric assay with a different substrate peptide (DYRKtide) (Table 3). In spite of this unequivocal detection of active xDYRK1B in *Xenopus *oocytes, we failed to detect the immunopreciptated xDYRK1B on Western blots (data not shown), apparently because the immunodetection of xDYRK1B on Western blots was less sensitive than the assay of its catalytic activity.

**Table 3:** xDYRK1B kinase activity in *Xenopus laevis *oocytes

	Phosphorylated peptide (pmol)				
	
IP (antibody)	ELISA-based assay (n = 3)				Radiometric assay (n = 1)
		
	1	2	3	Mean ± S.D.	
**Pre-immune**	0.30	1.23	1.27	0.93 ± 0.55	0.15
**αDYRK1B**	6.19	8.19	11.34	8.57 ± 2.60	3.37

Immunocomplex kinase assays were performed with lysates of about 100 μl settled oocytes per assay. Results of three independent ELISA assays (1-3) with tau_207-219 _and one radiometric assay with DYRKtide are reported.

## Discussion

In the present report we describe a simple and non-radiometric assay sufficiently sensitive to measure endogenous activities of DYRKs in cell and tissue lysates. We demonstrate the usefulness of the method by measuring the activity of DYRK1A in mouse heart lysate and of a novel DYRK1B-related kinase in *Xenopus laevis *oocytes.

The novel assay relies on the detection of the phosphorylated substrate peptide by a phosphospecific antibody in a direct sandwich ELISA configuration. Existing phospho-ELISA procedures for *in vitro-*kinase assays have been designed mainly for screening application of protein kinase inhibitors, where recombinant kinases are used and sensitivity is not a major issue (*e.g. *[7,8,10,27], as well as many commercial assays). In our ELISA configuration, we use the phospho-specific antibody as a capturing antibody. This set-up has already been utilized for the detection of phosphoproteins in cellular lysates, where the sensitive detection of a small fraction of phosphoprotein is a key issue [5]. Here we show that this configuration allows the detection of small amounts (10-100 fmol) of phosphopeptide in reaction mixtures containing a great excess of the unphosphorylated substrate. The inverse configuration of the ELISA, in which the biotinylated peptide was bound to streptavidin-coated wells, was much less sensitive with a detection limit of about 1 pmol phosphorylated peptide (Figure 1D). We have not further investigated the reason for this difference, but we speculate that the high density of the biotinylated peptide that is immobilized by the streptavidin, as compared with the few phosphorylated molecules that are captured by the phosphoantibody, impedes the binding of the detection antibody to the epitope. Further analysis of other peptide/antibody pairs will be necessary to determine whether this difference between the two configurations of the ELISA is generally valid.

Advantages of our method in comparison with other kinase assays are i) the high sensitivity ii) the possibility to use high ATP levels, iii) the avoidance of radioisotopes and iv) the simple set-up, *i.e. *the independence of specialized reagents (such as labelled tracers) or instruments. Apart from the plate reader, the method basically requires a high-quality phosphospecific antibody and a biotinylated substrate peptide. A drawback of ELISAs in comparison with radiometric assays is the semiquantitative nature with respect to the determination of the amount of phosphate incorporated into substrate. However, even radiometric assays of kinase activities are only rarely evaluated in a quantitative manner, because they frequently aim at the comparison of kinase activities in different samples analysed in parallel. In addition, the ELISA allows for the quantification of the DYRK activity in a given lysate by relating samples to a phosphopeptide standard. A minor limitation of the ELISA is the narrow linear range of detection, which can make it necessary to determine a suitable dilution of the reaction mixes.

We show that the present assay with tau_207-219 _as the substrate peptide can also be employed for measuring the activity of DYRK2, a kinase with 46% sequence identity with DYRK1A in the catalytic domain. Thr212 in tau has also been reported to be phosphorylated by other kinases such as protein kinase A (PKA) and cyclin-dependent kinase 5 (CDK5) [28,29]. This lack of specificity precludes the use of this particular substrate for assays of a specific kinase in cell lysates, but on the other hand should allow its use for PKA and CDK5 in immunocomplex kinase assays. Obviously, the method should be applicable for other protein kinases, provided that suitable substrate peptide sequences are known and that the respective phosphoepitope-specific antibodies are available. It should be noted that the quality of the phosphospecific antibody is a critical issue, because any binding of the unphosphorylated peptide will directly affect the background and thereby limit sensitivity.

The evolutionary conservation of the DYRK family encouraged us to assess whether the ELISA could be used for measuring the activity of non-mammalian DYRKs. Given that in many species DYRKs are involved in differentiation processes [11-13,30-33], early *Xenopus *development is an interesting model system to study the role of DYRKs in vertebrate embryogenesis. Here we have for the first time determined the activity of a kinase related to mammalian DYRK1B (designated xDYRK1B) in *Xenopus laevis *oocytes. In a previous report, an mRNA for a DYRK1-releated kinase has been identified in a microarray experiment with mRNA from *Xenopus laevis *oocytes and been designated "DYRK1A" [14]. However, the probe on the array was derived from a *Xenopus tropicalis *expressed sequence tag (TNeu006j18, GenBank acc. AL635205) that is derived from the Dyrk1a.2 gene, which is more closely related to DYRK1B (see Table 2). Surprisingly, Qu et al. [14] used a polyclonal antibody directed against the C-terminus of human DYRK1A to detect a 90-kD band in lysates of matured oocytes. These results may suggest that both xDYRK1A and xDYRK1B are expressed in *Xenopus *oocytes. Our attempts to immunoprecipitate xDYRK1A from the oocyte lysates remained unsuccessful (data not shown), but it is possible that the monoclonal anti human DYRK1A antibody that we used (from Abnova, Taipei, Taiwan) does not recognize xDYRK1A. Further studies will be necessary to dissect the roles of xDYRK1A and xDYRK1B in *Xenopus *development.

## Conclusion

The use of a phosphospecific capture antibody in ELISA-based kinase assays allows for the sensitive measurement of endogenous DYRK activity in diverse biological samples. With the help of this method, kinase activity of DYRK1B was detected in *Xenopus laevis *oocytes. We expect that this assay should prove useful for the study of kinase functions in various model systems.

## Methods

### Peptides and antibodies

A biotinylated substrate peptide mimicking the sequence around Thr212 of the tau protein (Biotin-Ttds-GSRSRTPSLPTPP-NH_2_, Thr212 is underlined, where Ttds is a proprietary linker) was selected from the Single Kinase Substrate Peptides commercially available from JPT Peptide Technologies (Berlin, Germany). For use in standard curves, a phosphorylated version was custom synthesized by Eurogentec (Seraing, Belgium). The optimized substrate peptide DYRKtide and the rabbit antibody directed against the C-terminus of human DYRK1B have been described previously [26,34]. The following antibodies were obtained from commercial sources: rabbit anti TAU(pT212) antibody (Cat. No. 44-740G; Invitrogen, Carlsbad, CA), mouse monoclonal antibodies for phosphotyrosine (PY99, Santa Cruz Biotechnology, Santa Cruz, CA), FLAG epitope (FLAG-M2, Sigma, Munich, Germany) and DYRK1A (clone 7D10; Abnova, Taipei City, Taiwan), and goat GFP-specific antibody (Rockland, Gilbertsville, PA).

### Preparation of mouse heart and *Xenopus *oocyte lysates

Hearts from TW-104 mice were weighed and 2 ml of lysis buffer (50 mM Tris-HCl pH7.5, 150 mM NaCl, 0.5% NP-40, 15% glycerol, 1 mM EDTA, 1 mM NaF, 1 mM Na_3_VO_4_, 10 μg/ml aprotinin, 10 μg/mL pepstatin, 10 μg/mL leupeptin, 1 μL PMSF) were added per 400 mg of tissue. The hearts were cut into small pieces, subjected to 10 strokes of medium rpm (stage 6 out of 10) in a Potter Elvehjem homogenizer, and the homogenates were then sonified for 20 s. After centrifugation (21,000 ***g***, 4°C, 10 minutes), the supernatant was taken and stored at -80°C. The lysates were cleared by pre-incubation for 30 minutes with 30 μL protein G agarose (EZview™ Red Protein G Affinity Gel, Sigma, Munich, Germany) and subsequent centrifugation before the supernatant was taken for immunoprecipitations. Follicle cell-free oocytes of oogenesis stages V or VI from *Xenopus laevis *[35] were kindly provided by the members of the Schmalzing group in the Institute of Pharmacology. The oocytes were lysed in 4 volumes of lysis buffer by up-and-down pipetting, and the lysates were cleared by repeated centrifugation to remove lipids and insoluble debris.

### Immunoprecipitation

For the immunoprecipitation from heart lysates, 1 μg monoclonal anti DYRK1A or 1 μg anti-FLAG antibody were added to a volume of lysate containing 1 mg of total protein. For the oocyte experiments, the amount of lysate derived from a volume of 100 μl settled oocytes was used for immunoprecipitation with either 1 μg anti DYRK1A antibody, 1 μl anti DYRK1B serum [26] or 1 μl non-reacting serum (preimmune serum of the same rabbit used to raise the DYRK1B-specific serum).

Immuncomplexes were captured by over-night incubation with protein G agarose at 4°C in an end-over-end rotator. After washing the agarose beads twice with TBS (50 mM Tris-HCl pH 7.5, 150 mM NaCl, 2 mM EDTA) supplemented with 0.1% Igepal CA-630, twice with TBS without Igepal and once with kinase buffer, bound immunocomplexes were assayed for *in vitro*-kinase activity (see below). In some experiments, the affinity matrix was divided equally between two test tubes during the last washing step to allow for the direct comparison of radioactive and non-radioactive assay.

### Cell culture and transfection

HeLa cells were cultured in Quantum 101 Medium for HeLa cells (PAA Laboratories, Pasching, Austria) and COS7 cells in DMEM medium supplemented with 10% fetal calf serum (PAA) at 37°C in a humidified 5% CO_2 _atmosphere. Transient transfections were performed using the FuGENE HD transfection reagent (Roche Molecular Biochemicals, Mannheim, Germany). Expression clones for GFP-DYRK1A and GFP-DYRK1B (p69 splicing variant) have been described previously [26,36]. An expression clone for untagged full length xDYRK1B (IMAGE clone 4724858) was obtained from Imagenes (Berlin, Germany).

### Western blotting

After washing the immunoprecipitates with TBS, bound proteins were eluted with Laemmli sample buffer, separated by SDS-PAGE (7%) and blotted onto nitrocellulose membranes. Immunodetection with commercial antibodies was performed according to the supplier's instructions. The DYRK1B antiserum was used at a dilution of 1:5000 for 2 h at room temperature, and the TBS for the washes following incubation with the primary antibody was supplemented with 0.1% SDS to reduce non-specific binding [26]. Western blots were developed using horseradish peroxidase-coupled secondary antibodies and chemiluminescence detection. The Clean-Blot IP Detection Reagent (Thermo Scientific, Rockford, IL) was used as a secondary antibody when proteins were immunodetected with the same antibody used for immunoprecipitation.

### Kinase assays

Recombinant glutathione S-transferase (GST) fusion proteins of rat DYRK1A-ΔC and human DYRK2 were prepared as described previously [34,36]. One unit of kinase activity was defined as the amount of enzyme that catalyzed the phosphorylation of 1 nmol DYRKtide per min at 30°C in kinase buffer (25 mM Hepes, pH 7.0, 5 mM MgCl_2_, 0.5 mM dithiothreitol).

To determine the assay sensitivity (Figure 2), reactions were performed in a total volume of 10 μl with variable amounts of the recombinant kinase, 50 μM tau_207-219 _and 100 μM ATP at 30°C for 30 min. Reactions were stopped by addition of 10 μl 10 mM EDTA and stored at -20°C until analysis by ELISA.

To prepare phosphorylated tau_207-219 _for the use in the ELISA (Figure 1), we aimed to achieve maximal phosphorylation by incubating 2.5 nmol tau_207-219 _with an excess of active kinase (4 mU/μL GST-DYRK1A-ΔC) in the presence of 0.82 mM ATP. The reaction mixture was incubated for 2 hours, and the same amount of kinase was supplemented after one hour. Total incorporation of phosphate was determined in a parallel reaction that was incubated in the presence of [γ-^33^P]ATP under identical conditions. All other kinase assays were incubated for 30 min at 30°C.

Immunocomplex kinase assays were carried out in a total reaction volume of 50 μl in kinase buffer, 100 μM ATP and 50 μM tau_207-219 _at 30°C for 30 min and stopped by addition of 50 μL 10 mM EDTA. Samples were stored at -20°C until evaluation by ELISA. Radiometric assays were run in the presence of [γ-^33^P]ATP (~ 2000 cpm/pmol). Incorporation of ^33^P into the biotinylated tau peptide was determined by spotting aliquots of the reaction mix onto a streptavidin membrane (SAM^2 ^Biotin Capture Membrane, Promega, Mannheim, Germany), which was then washed as described by the manufacturer and evaluated by scintillation counting. Radiometric immunocomplex kinase assays with the substrate peptide DYRKtide (at 100 μM) were run with 10 μM ATP as described before [37].

### Direct Sandwich ELISA

MaxiSorp F96 MicroWell™ Plates (Nunc, Langenselbold, Germany) were coated with 100 ng/well TAU(pT212) antibody at 4°C overnight. To keep the antibody consumption low, we used 100 ng/well of capturing antibody for coating in the standard protocol, although 200 ng/well gave a slightly higher readout (1.2-fold). After blocking for 1 hour with 200 μl/well 2% (w/v) bovine serum albumin (fraction V, modified Cohn, Calbiochem) in phosphate buffered saline (PBS) (140 mM NaCl, 3 mM KCl, 8 mM Na_2_HPO_4_, 1.8 mM KH_2_PO_4_, pH 7.4), the antigen was added in a volume of 100 μL/well and the plates were incubated for 2 h at room temperature. Bound antigen was detected with streptavidin-HRP conjugate (Pierce, Rockford, IL; 100 μL/well, 2 h at room temperature; 25 ng/ml) and subsequent incubation with 100 μL/well TMB (3,3',5,5'-tetramethylbenzidine) substrate solution until colour development was observed. Other detection reagents (HRP-linked anti-biotin antibody from CST; strepavidin-HRP from R&D systems) were less sensitive, and higher concentration of the streptavidin-HRP conjugate resulted in higher background in empty wells. The reaction was then stopped by adding 50 μl/well 1 M HCl, and plates were read at 450 nm (reference wavelength 620 nm). After each incubation step, plates were washed 4 times with PBS, pH 7.4, 0.1% Tween-20. Antibody and antigen were diluted in PBS.

### ELISA format based on streptavidin-coated plates

Reacti-Bind™ Streptavidin High Binding Capacity Coated 96-Well Plates (Pierce; Rockford, IL, USA) were loaded with the indicated concentrations of the biotinylated peptides (100 μL/well) and incubated at RT for 2 h. After washing, bound phosphopeptide was detected with rabbit anti TAU(pT212) antibody (1:16.000) and HRP-linked goat anti rabbit IgG (Fc) (Pierce) (1:40.000) and TMB substrate as described above. PBS with 2% BSA and 0.1% Tween-20 was used for plate washing and for dilution of antigen and antibodies.

### Data evaluation and curve fitting

Standard curves of phosphorylated tau peptide and the graphical representation of the time course shown in Figure 4 were generated by non-linear curve fitting using GraphPad Prism 5 (GraphPad Software, La Jolla, CA, USA).

## Authors' contributions

EL designed and carried out most of the experiments and analysed the results. KK carried out the experiments with *Xenopus *DYRK1. WB conceived of and coordinated the study and wrote the manuscript. All authors read and approved the final manuscript.

## Supplementary Material

Additional file 1 **Figure S1**. This PDF file contains a supplementary figure that shows a multiple sequence alignment of human and *Xenopus laevis *DYRK1 isoforms. Click here for file
