# Supplementary material for: Development of a sensitive non-radioactive protein kinase assay and its application for detecting DYRK activity in Xenopus laevis oocytes
Source: BMC Biochem. 2010 May 20;11:20. doi: 10.1186/1471-2091-11-20 (PMC2885986; doi:10.1186/1471-2091-11-20)
Supplement: Additional file 1 — Figure S1. This PDF file contains a supplementary figure that shows a multiple sequence alignment of human and Xenopus laevis DYRK1 isoforms. [file 1471-2091-11-20-S1.PDF]

```
hDYRK1A          MHTGGETSACKPSSVRLAPSFSFHAAGLQMAGQMPHSH-QYSDRRQPNIS
xDYRK1A          MHTGGETSACKPSSVRLAPSFSFHAAGLQMAGQMSHSHQQYSDRHQQNLN
xDYRK1B                                            MSSQHSHAPFSSLQSMADH
hDYRK1B                                            MAVPPGHGPFSGFPGPQEH

hDYRK1A          DQQVSALSYSDQIQQPLTNQVMPDIVMLQRRMPQTFRDPATAPLRKLSVD
xDYRK1A          DQQASALPYNDQTPQPLPN---------QRRMPQTFRDPATAPLRKLSVD
xDYRK1B          ------------------QQGLPDMTILQRRIPLTFRESAAAPLRKLSVD
hDYRK1B          ------------------TQVLPDVRLLPRRLPLAFRDATSAPLRKLSVD

hDYRK1A          LIKTYKHINEVYYAKKKRRHQQGQGDDSSHKKERKVYNDGYDDDNYDYIV
xDYRK1A          LIKTYKHINEVYYAKKKRRHQQGQGDDSSHKKERKVYNDGYDDDNYDYIV
xDYRK1B          LIKTYKHINEVYYTKKKRRAQQVPPEDSSTKKERKVFNDGFDDDNYDYIV
hDYRK1B          LIKTYKHINEVYYAKKKRRAQQAPPQDSSNKKEKKVLNHGYDDDNHDYIV

hDYRK1A          KNGEKWMDRYEIDSLIGKGSFGQVVKAYDRVEQEWVAIKIIKNKKAFLNQ
xDYRK1A          KNGEKWMDRYEIDSLIGKGSFGQVVKAYDRVEQEWVAIKIIKNKKAFLNQ
xDYRK1B          KNGEKWMDRYEIDSLIGKGSFGQVVKAYDHHDQEWVAIKIIKNKKAFLNQ
hDYRK1B          RSGERWLERYEIDSLIGKGSFGQVVKAYDHQTQELVAIKIIKNKKAFLNQ

hDYRK1A          AQIEVRLLELMNKHDTEMKYYIVHLKRHFMFRNHLCLVFEMLSYNLYDLL
xDYRK1A          AQIEVRLLELMNKHDTEMKYYIVHLKRHFMFRNHLCLVFEMLSYNLYDLL
xDYRK1B          AQIELRLLELMNKHDTEMKYYIVHLKRHFMFRNHLCLVFELLSYNLYDLL
hDYRK1B          AQIELRLLELMNQHDTEMKYYIVHLKRHFMFRNHLCLVFELLSYNLYDLL

hDYRK1A          RNTNFRGVSLNLTRKFAQQMCTALLFLATPELSIIHCDLKPENILLCNPK
xDYRK1A          RNTNFRGVSLNLTRKFAQQMCTALLFLATPELSIIHCDLKPENILLCNPK
xDYRK1B          RNTNFRGVSLNLTRKFAQQLCTALLFLATPELSIIHCDLKPENILLCNPK
hDYRK1B          RNTHFRGVSLNLTRKLAQQLCTALLFLATPELSIIHCDLKPENILLCNPK

hDYRK1A          RSAIKIVDFGSSCQLGQRIYQYIQSRFYRSPEVLLGMPYDLAIDMWSLGC
xDYRK1A          RSAIKIVDFGSSCQLGQRIYQYIQSRFYRSPEVLLGTPYDLAIDMWSLGC
xDYRK1B          RSAIKIVDFGSSCQLGQRIYQYIQSRFYRSPEVLLGMPYDLAIDMWSLGC
hDYRK1B          RSAIKIVDFGSSCQLGQRIYQYIQSRFYRSPEVLLGTPYDLAIDMWSLGC

hDYRK1A          ILVEMHTGEPLFSGANEVDQMNKIVEVLGIPPAHILDQAPKARKFFEKLP
xDYRK1A          ILVEMHTGEPLFSGANEVDQMSKIVEVLGIPPAHILDQAPKARKFFEKMP
xDYRK1B          ILVEMHTGEPLFSGSNEVDQMNKIVEVLGTPPNHMLDQAPKARKYFDKLP
hDYRK1B          ILVEMHTGEPLFSGSNEVDQMNRIVEVLGIPPAAMLDQAPKARKYFERLP

hDYRK1A          DGTWSLKKTKDGKREYKPPGTRKLHNILGVETGGPGGRRAGESGHTVADY
xDYRK1A          EGTWNLKKTKDGKKEYKPPGTRKLHNILGVENGGPGGRRAGESGHTVADY
xDYRK1B          EGTWTVKKNKDLKKDYKVPGTRRLHEVLGVETGGPGGRRGGEQGHSPSDY
hDYRK1B          GGGWTLRRTKELRKDYQGPGTRRLQEVLGVQTGGPGGRRAGEPGHSPADY

hDYRK1A          LKFKDLILRMLDYDPKTRIQPYYALQHSFFKKTADEGTNT---SNSVSTS
xDYRK1A          LKFKDVILRMLDYDAKTRIQPYYALQHSFFKKTADEGTNT---SNSVSTS
xDYRK1B          LKFKDLILRMLDYDPKTRITPFYALQHNFFKKTTDEGTNT---SNSNSTS
hDYRK1B          LRFQDLVLRMLEYEPAARISPLGALQHGFFRRTADEATNTGPAGSSASTS

hDYRK1A          PAMEQSQSSGTTSSTSSSSGGSSGTSNSGRARSDPTHQHRHSGGHFTAAV
xDYRK1A          PAMEQSQSSGTTSSTSSSSGGSSGTSNSGRARSDPTHQHRHSGGHF-TTA
xDYRK1B          PAMD-HSHSTSTTSSVSSSGGSSGSSNDNRNYRYSNRYYNSAV-------
hDYRK1B          PAPLDTCPSSSTASSISSSGGSSGSSSDNRTYRYSNRYCGGPGP------

hDYRK1A          QAMDCETHSPQVRQQFPAPLGWSGTEAPTQVT--------VETHPVQETT
xDYRK1A          VAMDCETHSPQVRQQFPP--GWTVPEAPTQVT--------IETHPVQETT
xDYRK1B          AHTDYEMQSPQTHSQQQIR-LWAGGDVPITNSDSYPQILPHKPTPSQPQH
hDYRK1B          PITDCEMNSPQVPPSQPLR-PWAGGDV------------PHKTHQAPASA

hDYRK1A          FHVAPQQNALHHHHGNSSHHHHHHHHHHHHHGQQALGNRTRPRVYNSPTN
xDYRK1A          FHVPPSQKNVPHHHGNGSHHHHHHHHHHHH--GQHILSNRTRTRIYNSPST
xDYRK1B          FHGNEPHHP-----------------------HPLYHHVNRPHYHRQLIT
hDYRK1B          SSLPGTGAQLPPQPRYLGRPPS----------------------------

hDYRK1A          SSSTQ--DSMEVGHSHHSMTSLSSSTTSSSTSSSSTGNQGNQAYQNRPVA
xDYRK1A          SSSTQ--DSMDIGNSHHSMTSLSSSTTSSSTSSSSTGNQGNQAYQNRPVA
xDYRK1B          SSSPQIPESMELS----------------------LGHRHPQSSSSLHP-H
hDYRK1B          PTSPPPPELMDVS--------------LVGGPADCSPPHPAPAP-QHPAA

hDYRK1A          ANTLDFGQNGAMDVNLTV--YSNPRQETGI--AGHP-TYQFSANTGPAHY
xDYRK1A          ANTLDFGQNGTLDVNLTV--YSNPRQETGI--TGHP-DYQYSANTGPGHY
xDYRK1B          HPSLDCTPFGSANLHLGVSAFRTRTVSGHTDSGVPLNYPYTSNTSS--M-
hDYRK1B          ------------------SALRTRMT------GGRP---PLPPPDDPA--

hDYRK1A          MTEGHLTMRQGADREESPMTGVCVQQSPVASS
xDYRK1A          VTEGQLTMRQGIDREDSPMTGVCVQQSPVASS
xDYRK1B          VGPPHI--RNRTETEESAMLGVCVPQSTAASS
hDYRK1B          -TLGP---HLG-------LRG--VPQSTAASS
```

Figure S1 - Sequence Alignment of human and Xenopus DYRK1A and DYRK1B

The catalytic domain is boxed. The bipartite nuclear localization signal in the N-terminal domain is highlighted in green. The histidine repeat in the C-terminal domain is domain is marked yellow.
